# Predictive value of combined MHR and Lp(a) for in-stent restenosis in coronary heart disease patients: a study based on GEE model

**DOI:** 10.3389/fcvm.2025.1672158

**Published:** 2026-01-12

**Authors:** Sijia Tu, Mengyang Cai, Gang Wang, Zhi Zhang

**Affiliations:** 1 Department of Cardiology, First People’s Hospital of Linping District, Hangzhou, Zhejiang, China; 2 School of Pharmaceutical Science, Zhejiang Chinese Medical University, Hangzhou, Zhejiang, China

**Keywords:** coronary heart disease, GEE model, in-stent restenosis, lipoprotein(a), monocytes, monocyte-to-HDL ratio

## Abstract

**Objective:**

To investigate the associations of monocyte count, lipoprotein(a) [Lp(a)], and monocyte-to-HDL ratio (MHR) with in-stent restenosis (ISR) in coronary heart disease (CHD) patients after drug-eluting stent (DES) implantation, and to develop a predictive risk model.

**Methods:**

This study enrolled 190 CHD patients who underwent DES implantation from 2019 to 2024. Based on 1-year coronary angiography, patients were divided into an ISR group (*n* = 40) and a Non-ISR group (*n* = 150). Baseline characteristics, laboratory markers, coronary lesions, and stent parameters were analyzed. Logistic regression and generalized estimating equation (GEE) models were used to identify independent predictors. ROC curves assessed the diagnostic performance. A risk score was constructed and its association with major adverse cardiovascular events (MACE) evaluated.

**Results:**

Compared to the Non-ISR group, ISR patients had higher monocyte count, MHR, and Lp(a) levels (all *P* < 0.05), and more frequent left main and multivessel disease. Monocyte count (OR = 1.949), Lp(a) (OR = 1.22), and MHR (OR = 1.009) were independent risk factors for ISR. ROC analysis showed AUCs of 0.815, 0.672, and 0.726 for monocytes, Lp(a), and MHR, respectively. Combined detection of MHR and Lp(a) improved the AUC to 0.860. The risk score effectively stratified patients, with a higher MACE incidence in the high-risk group (35% vs. 10%).

**Conclusion:**

Monocyte count, Lp(a), and MHR are closely linked to ISR in CHD patients post-DES. Combined assessment enhances risk prediction, offering a basis for early identification and personalized management to reduce restenosis and improve outcomes.

## 1 Introduction

Coronary heart disease (CHD) is characterized by coronary artery stenosis or obstruction due to atherosclerosis, leading to myocardial ischemia, hypoxia, or even necrosis, which can result in severe cardiac events (1). Global epidemiological data indicate that CHD has become one of the leading causes of death worldwide, responsible for over seven million deaths annually, with the highest morbidity and mortality rates observed in Europe, North America, and other developed regions (2). Percutaneous coronary intervention (PCI), known for its minimal invasiveness, rapid recovery, and fewer complications, has emerged as the primary treatment modality for CHD since its introduction in 1979, significantly reducing patient mortality rates. However, PCI has inherent limitations, and in-stent restenosis (ISR) remains a critical issue, adversely affecting the long-term effectiveness and clinical prognosis of treated patients (3).

ISR is typically defined as the development of new lesions or vessel stenosis of ≥50% within the stent or its margins (within 5 mm), leading to restricted coronary blood flow and potentially triggering acute coronary syndrome (ACS), which poses a serious threat to patient survival (4). Although the advent of drug-eluting stents (DES), with their antiproliferative drug coatings, has significantly reduced ISR rates, it remains clinically relevant. This underscores the ongoing need for a deeper understanding of the mechanisms underlying ISR and the identification of early predictive indicators. Previous studies have identified vascular smooth muscle cell proliferation, endothelial injury, and persistent vascular inflammation as key pathological processes involved in ISR (5–7). Among these, inflammation is considered the central driving force. Monocytes play a pivotal role in mediating inflammatory responses in both atherosclerosis and ISR by interacting with endothelial cells and platelets, thereby initiating vascular inflammation, endothelial dysfunction, and thrombus formation (8, 9). Conversely, high-density lipoprotein cholesterol (HDL-C) exerts anti-inflammatory and monocyte chemotaxis-inhibiting effects, providing a protective role against ISR (10, 11). Lipoprotein(a) [Lp(a)], a specific subtype of low-density lipoprotein, contributes to ISR by promoting intimal deposition, accelerating atherosclerosis progression, and enhancing thrombogenesis (12). Recently, the monocyte-to-HDL-C ratio (MHR) has been proposed as a novel integrated marker reflecting chronic inflammation and dysregulated lipid metabolism. Evidence suggests that MHR closely correlates with CHD severity and adverse prognosis, potentially outperforming either monocyte count or HDL-C level alone (13, 14). Elevated MHR has already been recognized as an independent risk factor for ISR following DES implantation in patients with acute myocardial infarction (15). Meanwhile, increased Lp(a) levels have been consistently associated with elevated risks of cardiovascular events, including CHD and stroke (16–18), although its specific role in ISR remains incompletely understood. Current evidence on MHR and Lp(a) in predicting ISR is predominantly derived from small-scale, single-center retrospective studies, lacking a systematic evaluation of their combined predictive value. Additionally, insufficient control of potential confounders and a lack of external validation have limited the clinical applicability and generalizability of these findings.

Currently, there is a lack of simple, accessible, blood-based tools [such as MHR and Lp(a)] to predict ISR risk. For patients undergoing DES implantation, early identification of high-risk individuals for ISR using routine clinical parameters remains a critical unresolved issue in clinical practice. This study systematically evaluated the associations between monocyte count, HDL-C, Lp(a), and MHR with ISR using a generalized estimating equation (GEE) model. For the first time, we developed a risk-scoring system based on MHR and Lp(a). This scoring system aims to provide a convenient, economical, and generalizable early prediction tool for individualized management, ultimately reducing ISR occurrence and improving the long-term prognosis of CHD patients.

## 2 Methods

### 2.1 Study population and grouping

This study enrolled patients diagnosed with CHD who underwent DES implantation at the First People's Hospital of Linping District, Hangzhou, between 2019 and 2024. Based on 1-year coronary angiography follow-up, patients were categorized into two groups: the ISR group (*n* = 40), consisting of patients who developed ISR confirmed by coronary angiography, and the non-ISR group (*n* = 150), consisting of patients with no evidence of ISR during follow-up. All patients provided written informed consent.

**Inclusion criteria:** (1) patients aged between 18 and 79 years with a diagnosis of CHD; (2) patients who underwent DES implantation and completed coronary angiography follow-up within one year post-procedure; (3) patients with at least one-year clinical follow-up and complete medical records. ISR diagnosis was confirmed if coronary angiography showed ≥50% stenosis in the stent or within 5 mm of its proximal or distal edges, with at least one of the following: recurrent angina, objective evidence of ischemia (e.g., ECG changes), fractional flow reserve (FFR) <0.80, intravascular ultrasound (IVUS) showing a minimum lumen area <4 mm^2^ (<6 mm^2^ for left main coronary artery), or asymptomatic patients with IVUS revealing lumen reduction ≥70%.

**Exclusion criteria:** patients were excluded if they had any of the following conditions: recent treatment with anti-inflammatory or chemotherapy medications; severe liver dysfunction (ALT > 3 times the upper limit of normal); severe renal dysfunction (eGFR < 30 mL/min/1.73m^2^); familial hypercholesterolemia; heart failure (left ventricular ejection fraction <45%); thyroid dysfunction; concurrent infectious diseases (e.g., chronic obstructive pulmonary disease, tuberculosis, pneumonia, upper respiratory infections, acute or chronic gastroenteritis, cholecystitis, appendicitis, pancreatitis, inflammatory bowel disease, gastric ulcer, myocarditis, pericarditis, or infective endocarditis); allergic reactions; autoimmune diseases; malignancy; pregnancy; congenital heart disease; history of coronary artery bypass grafting (CABG); or coronary anomalies (e.g., vessel tortuosity, malformation, aneurysm, or dissection). This study was reviewed and approved by the Ethics Committee of the First People's Hospital of Linping District, Hangzhou (Approval Number: 2023-Research-230).

### 2.2 Baseline characteristics and laboratory measurements

Baseline clinical data were systematically collected from all enrolled patients, including demographic information [sex, age, height, weight, and body mass index (BMI)], comorbidities (hypertension, diabetes mellitus, hyperlipidemia, smoking history, alcohol consumption), clinical symptoms (chest pain, dyspnea, syncope), lifestyle factors (physical activity, dietary habits, and sleep quality), and medication history (antihypertensive drugs, antidiabetic medications, statins, antiplatelet drugs). Blood samples were collected to measure various hematological, biochemical, inflammatory, cardiac, and lipid parameters, including white blood cell count, monocyte count, neutrophil count, lymphocyte count, platelet count, hemoglobin levels, platelet distribution width (PDW), mean platelet volume (MPV), red cell distribution width (RDW), HDL-C, low-density lipoprotein cholesterol (LDL-C), Lp(a), glycated hemoglobin (HbA1c), cardiac troponin I (cTnI), N-terminal pro-B-type natriuretic peptide (NT-proBNP), uric acid (UA), serum creatinine (Scr), fibrinogen, D-dimer, and C-reactive protein (CRP). Additionally, physiological parameters such as heart rate, blood pressure, and ejection fraction (EF) were recorded.

### 2.3 Coronary angiography analysis

Coronary angiography results were independently reviewed and interpreted by three experienced cardiologists, each holding a position of associate chief physician or higher, to ensure diagnostic accuracy and consistency. The primary evaluations included identifying lesion segments, lesion types, degree of stenosis, and other coronary artery characteristics. Lesion segments were defined by determining the specific location and extent of the lesions via angiography, with detailed assessments of involvement in the main coronary arteries and their branches. The severity of stenosis was visually assessed and categorized as mild (≤50%), moderate (51%–75%), or severe (>75%), with IVUS used when necessary for supplementary evaluation. For stent-related lesions, special attention was given to stenosis within the stent and its proximal and distal segments to determine the presence of ISR. Additionally, for patients who underwent DES implantation, stent placement characteristics, including apposition to the vessel wall, stent length, number of stents, and overlap configuration, were evaluated due to their close association with ISR occurrence.

### 2.4 Follow-up and outcome events

All enrolled patients were followed for one year through outpatient visits and telephone interviews. The primary endpoint was the occurrence of ISR, while secondary endpoints included major adverse cardiovascular events (MACE), such as all-cause mortality, recurrent ST-segment elevation myocardial infarction (STEMI), non-fatal stroke, malignant arrhythmia, and target vessel revascularization.

### 2.5 Statistical analysis

Initially, baseline characteristics such as sex, age, BMI, smoking history, diabetes mellitus, and hypertension were compared between ISR and non-ISR groups. For continuous variables, normality testing was performed using the Shapiro–Wilk test. Variables that followed a normal distribution were compared using *t*-tests, while non-normally distributed variables were appropriately transformed (e.g., log transformation). Categorical variables were compared using chi-square tests to exclude potential confounding factors. To further explore the independent associations between various biomarkers and ISR occurrence, GEE models were utilized, as this method effectively accounts for correlated patient data. In the multivariable analysis, to ensure the reliability and scientific rigor of the results, we first selected potential confounding variables based on the following criteria: (1) clinical characteristics and biomarkers potentially associated with ISR, selected based on a literature review and known ISR risk factors; (2) variables significantly associated with ISR (*P* < 0.05) identified through univariate analysis and included in the multivariable GEE model; (3) clinically relevant variables that were not significantly associated with ISR in the univariate analysis (e.g., age, sex) were included based on expert review and clinical judgment; (4) the model was optimized using stepwise regression to ensure that the included variables had statistical significance. To preserve more information, future studies may consider analyzing these biomarkers as continuous variables, such as using splines or per standard deviation increase, rather than dichotomizing them based on median values. Additionally, they could be divided into tertiles or quartiles to further enhance statistical power and reduce information loss. In this study, we categorized the biomarkers [such as MHR and Lp(a)] into high and low groups based on the sample median and compared their relationship with ISR occurrence. Additionally, biomarkers were categorized into high and low levels based on the median value of the sample to assess their relationship with ISR occurrence. For MHR and Lp(a), the optimal cutoff values were first determined using receiver operating characteristic (ROC) curves. Specifically, the ROC curve was used to identify the threshold that maximized the balance between sensitivity and specificity, thereby maximizing prediction accuracy. Based on this, the maximum Youden index method was applied to determine the optimal cutoff values for MHR and Lp(a), which were then used to categorize these biomarkers into high and low levels for further analysis of their predictive ability for ISR occurrence. ROC curves were plotted to assess the diagnostic value of Lp(a), monocyte count, and MHR in predicting ISR, and the area under the curve (AUC) was used to evaluate their sensitivity and specificity. To further enhance the reliability of the risk score generation, we validated the regression coefficients. Specifically, internal validation and resampling methods were used to support the regression coefficients used in the model. Through k-fold cross-validation and bootstrap resampling, we evaluated the stability and accuracy of the regression coefficients, ensuring the consistency and reliability of the risk score model across different datasets. Although the ROC analysis in this study showed relatively robust results, the width of the AUC confidence intervals may have been influenced by the small sample size. Therefore, we performed a sample size estimation in our analysis to ensure sufficient statistical power to support our conclusions. Future studies with larger sample sizes for validation will further enhance the credibility and broader applicability of the results. Finally, univariate and multivariate regression analyses were performed to identify independent risk factors for ISR. All statistical analyses were conducted using SPSS version 27.0.

## 3 Results

A total of 190 patients (118 males and 72 females) were enrolled in this study. Based on follow-up coronary angiography, patients were divided into two groups: the non-ISR group (*n* = 150) and the ISR group (*n* = 40). No statistically significant differences were found between the two groups in terms of baseline characteristics, including sex, age, diabetes mellitus, hypertension, smoking history, alcohol consumption, and family history (all *P* > 0.05). Laboratory results revealed significantly higher white blood cell and monocyte counts in the ISR group compared to the non-ISR group (*P* = 0.012 and *P* = 0.001, respectively). Biochemical indicators also showed significantly elevated HDL-C, Lp(a), and MHR levels in the ISR group compared to the non-ISR group (*P* = 0.022, *P* = 0.0267, and *P* = 0.003, respectively). However, no significant differences were observed between the two groups in other parameters, including lipid profiles and renal function markers (*P* > 0.05). Detailed data are presented in Table 1.

**Table 1:** Baseline characteristics of patients [*n* (%), mean ± SD].

Variables	Non-ISR group (*n* = 150)	ISR group (*n* = 40)	*χ*^2^/*Z*/*t*	*P* value
Male [*n*] (%)	92 (61.33%)	26 (65%)	0.180	0.671
Hypertension [*n*] (%)	104 (69.33%)	35 (87.5%)	5.307	0.054
DM [*n*] (%)	47 (31.33%)	11 (27.5%)	0.219	0.640
Smoking [*n*] (%)	124 (82.67%)	32 (80%)	0.153	0.696
Alcohol [*n*] (%)	115 (76.67%)	32 (80%)	0.200	0.654
Family history [*n*] (%)	21 (14%)	5 (12.5%)	0.060	0.806
Age (years)	65.22 ± 9.72	63.27 ± 10.17	0.674	0.508
BMI (kg/m^2^)	25.18 ± 5.02	23.41 ± 4.17	1.343	0.176
WBC (×10^9^/L)	6.83 ± 0.30	7.45 ± 0.36	2.389	0.012
Neutrophils (×10^9^/L)	4.51 ± 0.64	4.70 ± 0.32	2.412	0.058
Lymphocytes (×10^9^/L)	1.65 ± 0.26	2.11 ± 0.63	2.573	0.351
Monocytes (×10^6^/L)	501.85 ± 7.96	641.72 ± 21.03	5.073	0.001
Platelets (×10^9^/L)	214.87 ± 4.23	217.26 ± 3.31	0.764	0.952
Hemoglobin (g/L)	133.90 ± 2.32	132.68 ± 3.19	2.804	0.734
PDW (fL)	14.49 ± 0.21	15.63 ± 0.34	0.873	0.661
MPV (fL)	8.31 ± 0.32	8.54 ± 0.47	0.551	0.517
RDW (%)	13.98 ± 0.06	14.01 ± 0.25	0.706	0.353
Fibrinogen (g/L)	3.25 ± 0.56	3.37 ± 0.72	1.972	0.171
D-dimer (mg/L)	0.12 ± 0.53	0.13 ± 0.46	2.175	0.864
HbA1c (%)	6.48 ± 0.69	6.53 ± 0.74	2.683	0.562
Troponin I (μg/L)	0.50 ± 0.61	0.51 ± 0.82	1.565	0.869
NT-proBNP (ng/L)	467.42 ± 78.36	486.69 ± 97.45	0.451	0.063
UA (mmol/L)	341.98 ± 6.16	359.34 ± 10.57	3.782	0.703
Scr (μmol/L)	72.46 ± 5.43	69.86 ± 6.64	3.61	0.232
Albumin (g/L)	44.48 ± 1.19	43.64 ± 3.17	2.979	0.484
TC (mmol/L)	3.54 ± 1.02	3.49 ± 1.17	0.143	0.918
Triglycerides (mmol/L)	1.29 ± 0.64	1.49 ± 1.31	0.541	0.579
LDL-C (mmol/L)	2.19 ± 0.08	2.18 ± 0.24	0.987	0.332
HDL-C (mmol/L)	1.41 ± 0.35	1.22 ± 0.34	−2.419	0.022
Lp (a) (mmol/L)	164.82 ± 37.48	366.78 ± 79.39	2.314	0.0267
MHR	508.46 ± 19.32	620.54 ± 41.23	5.942	0.003
Heart rate (beats/min)	73.28 ± 2.287	71.65 ± 3.136	0.412	0.687
SBP (mmHg)	139.58 ± 3.445	131.25 ± 2.875	1.865	0.062
DBP (mmHg)	76.71 ± 2.633	76.83 ± 1.936	0.067	0.958
LVEF (%)	63.16 ± 2.291	62.76 ± 2.035	0.217	0.953

BMI, body mass index; DBP, diastolic blood pressure; DM, Diabetes mellitus; HbA1c, glycated hemoglobin; HDL-C, high-density lipoprotein cholesterol; Lp(a), lipoprotein(a); LDL-C, low-density lipoprotein cholesterol; LVEF, left ventricular ejection fraction; MHR, monocyte-to-HDL ratio; MPV, mean platelet volume; NT-proBNP, N-terminal pro-B-type natriuretic peptide; PDW, platelet distribution width; RDW, red cell distribution width; SBP, systolic blood pressure; Scr, serum creatinine; TC, Total cholesterol; UA, uric acid; WBC, white blood cell count.

The comparison of stent-related characteristics between the two groups is shown in Table 2. The average number of implanted stents was 1.86 ± 0.22 in the non-ISR group and 1.62 ± 0.23 in the ISR group, with no statistically significant difference (*P* = 0.31). Similarly, the stent length was slightly longer in the non-ISR group (33.03 ± 1.35 mm) compared to the ISR group (27.68 ± 1.76 mm), although this difference did not reach statistical significance (*P* = 0.19). Regarding mean stent deployment pressure, values were 7.36 ± 0.34 atm in the non-ISR group and 7.85 ± 0.96 atm in the ISR group. Despite a marginally higher pressure in the ISR group, the difference was not statistically significant (*P* = 0.15). Stent diameter was 5.67 ± 2.08 mm in the non-ISR group compared to 4.21 ± 1.43 mm in the ISR group, also showing no significant difference (*P* = 0.11). Collectively, these results indicate no statistically significant differences between the non-ISR and ISR groups in terms of stent number, length, deployment pressure, or diameter.

**Table 2:** Stent-related characteristics of patients [n (%), mean ± SD].

Variables	Non-ISR group (*n* = 150)	ISR group (*n* = 40)	*t*	*P* value
Number of stents (*n*)	1.86 ± 0.22	1.62 ± 0.23	0.87	0.31
Stent length (mm)	33.03 ± 1.35	27.68 ± 1.76	0.43	0.19
Stent deployment pressure (atm)	7.36 ± 0.34	7.85 ± 0.96	1.64	0.15
Stent diameter (mm)	5.67 ± 2.08	4.21 ± 1.43	1.16	0.11

Comparisons of SYNTAX scores and lesion characteristics are presented in Table 3. The ISR group had a slightly higher mean SYNTAX score compared to the non-ISR group (29.0 ± 5.2 vs. 24.2 ± 5.3), with borderline statistical significance (*P* *=* 0.054). In the stratified analysis of SYNTAX scores, no significant differences were observed between the two groups in terms of low (≤22), intermediate (23–32), or high (≥33) score distributions (*P* *=* 0.110, 0.286, and 0.299, respectively). The incidence of multivessel disease was slightly higher in the ISR group than in the non-ISR group (57.5% vs. 38.0%). Although this difference did not reach statistical significance (*P* *=* 0.076), it suggested a potential trend. Notably, the proportion of patients with left main coronary artery disease was significantly higher in the ISR group compared to the non-ISR group (22.5% vs. 9.3%, *P* *=* 0.023).

**Table 3:** SYNTAX scores and coronary lesion characteristics of patients [n (%), mean ± SD].

Variables	Non-ISR group (*n* = 150)	ISR group (*n* = 40)	χ2/Z/t	*P* value
SYNTAX score	24.2 ± 5.3	29.0 ± 5.2	−1.75	0.054
SYNTAX score category				
Low (≤22)	66 (44%)	12 (30%)	2.558	0.110
Intermediate (23–32)	72 (48%)	23 (57.5%)	1.140	0.286
High (≥33)	14 (9.3%)	6 (15%)	1.077	0.299
Multivessel disease [*n*] (%)	57 (38%)	23 (57.5%)	4.926	0.076
Left main disease [*n*] (%)	14 (9.3%)	9 (22.5%)	5.145	0.023

The results of the logistic regression analysis are presented in Table 4. Evaluation of various clinical and biochemical parameters revealed that NT-proBNP (OR = 2.055, 95% CI: 0.585–2.914, *P* = 0.826), HDL-C (OR = 0.178, 95% CI: 0.007–4.965, *P* = 0.311), and white blood cell count (OR = 1.057, 95% CI: 0.927–1.394, *P* = 0.089) were not significantly associated with ISR. While the association between SYNTAX score and ISR approached significance (*P* = 0.086), it did not reach the threshold for statistical significance, suggesting a limited relationship with ISR risk. Notably, although HDL-C exhibited an OR less than 1, indicating a potential protective trend, this finding was not statistically significant. In contrast, monocyte count, Lp(a), and MHR were significantly elevated in the ISR group and identified as independent risk factors for ISR. The OR for monocyte count was 1.949 (95% CI*:* 1.132–3.317, *P* *=* 0.017), indicating that elevated monocyte levels significantly increased ISR risk. Lp(a) showed an OR of 1.22 (95% CI*:* 1.02–1.48, *P* *=* 0.046), suggesting it as a potential risk factor. Furthermore, MHR had an OR of 1.009 (95% CI*:* 1.002–1.012, *P* *=* 0.032), demonstrating that higher MHR levels may contribute to increased ISR occurrence. These findings highlight the important roles of elevated monocyte count, Lp(a), and MHR in the development of ISR, providing valuable references for clinical risk monitoring and intervention.

**Table 4:** Logistic regression analysis of risk factors for in-stent restenosis after DES implantation.

Variables	OR	95% CI	*P* value
NT—proBNP (ng/L)	2.055	0.585–2.914	0.826
HDL-C (mmol/L)	0.178	0.007–4.965	0.311
SYNTAX score	1.018	0.913–2.392	0.086
WBC (×10^9^/L)	1.057	0.927–1.394	0.089
Monocytes (×10^6^/L)	1.949	1.132–3.317	0.017
Lp (a) (mmol/L)	1.22	1.02–1.48	0.046
MHR	1.009	1.002–1.012	0.032

HDL-C, high-density lipoprotein cholesterol; Lp(a), lipoprotein(a); MHR, monocyte-to-HDL ratio; NT-proBNP, N-terminal pro-B-type natriuretic peptide; WBC, white blood cell count.

The results of the GEE model analysis are presented in Table 5. Patients were divided into high and low groups for MHR and Lp(a) based on median values, with levels below the median classified as the low group and levels equal to or above the median classified as the high group. The GEE analysis revealed that patients in the high MHR group had a *β* coefficient of 2.482 with a standard error of 0.987 (95% CI*:* 1.083–3.386, *P* *=* 0.004), indicating that elevated MHR significantly increased the risk of ISR, with the low group serving as the reference (*β* = 0). Similarly, for Lp(a), the high-level group showed a *β* coefficient of 1.472 with a standard error of 0.224 (95% CI*:* 1.374–2.865, *P* *=* 0.009), demonstrating a significant association between higher Lp(a) levels and the occurrence of ISR. These results suggest that in patients undergoing DES implantation, elevated MHR and Lp(a) levels may serve as independent predictive markers for ISR.

**Table 5:** GEE model analysis of risk factors for in-stent restenosis after DES implantation.

Variables	*β*	SD (*β*)	95% CI	*P* value
Lp(a) high group^a^	1.472	0.224	1.374–2.865	0.009
MHR high group^a^	2.482	0.987	1.083–3.386	0.004

Lp(a), lipoprotein(a); MHR, monocyte-to-HDL ratio.

a Low-level group was used as the reference category.

**Figure 1:**
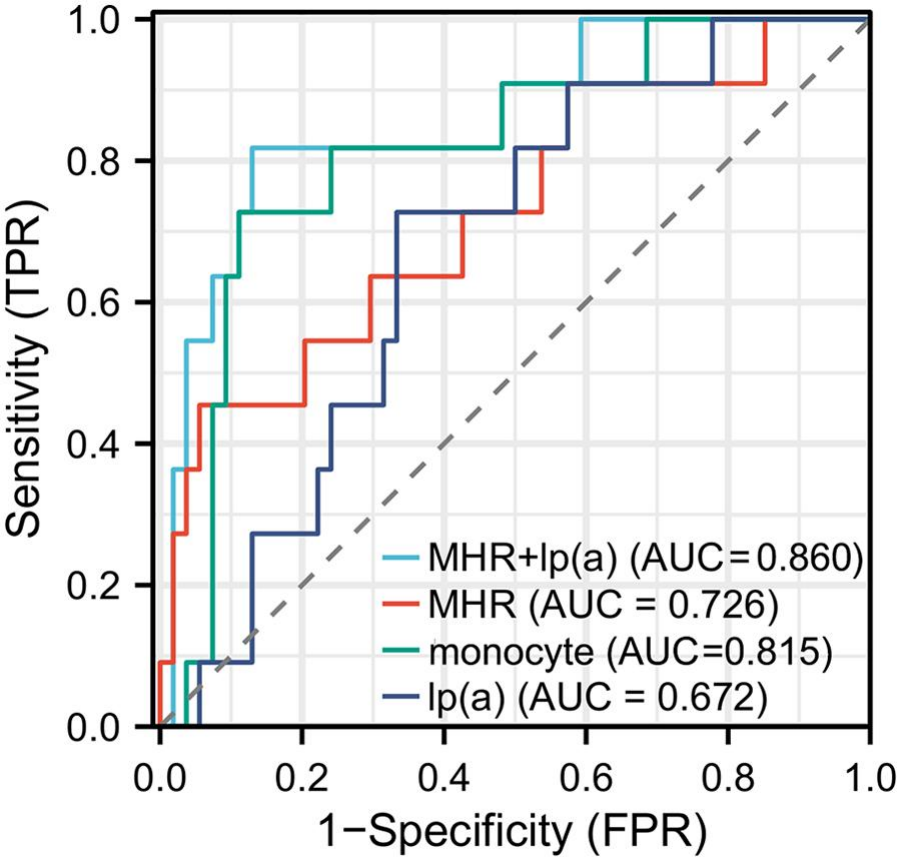
Receiver operating characteristic (ROC) curves for monocyte count, lipoprotein(a) [Lp(a)], MHR, and their combination in predicting in-stent restenosis (ISR).

ROC curve analysis was conducted to evaluate the potential predictive value of Lp(a), monocytes, and MHR for ISR occurrence (Figure 1). The results showed that Lp(a) exhibited moderate diagnostic value, with an AUC of 0.672 (95% CI*:* 0.517–0.827; *P* < 0.001), demonstrating good specificity (72.72%) and moderate sensitivity (66.67%). Although Lp(a) alone had relatively low sensitivity, its higher specificity suggests an advantage in correctly identifying patients without ISR. In contrast, MHR displayed better predictive capability, with an AUC of 0.726 (95% CI*:* 0.545–0.906; *P* < 0.001), specificity of 64.45%, and notably high sensitivity of 94.44%. This indicates that MHR is highly sensitive for screening high-risk patients, effectively identifying the majority of ISR cases, although its lower specificity implies a potential for false positives. Further analysis revealed that monocytes had an AUC of 0.815 (95% CI*:* 0.675–0.955; *P* < 0.001), with specificity and sensitivity of 72.72% and 88.89%, respectively. These findings suggest that monocytes possess strong diagnostic performance in assessing ISR risk, particularly due to their high sensitivity, enabling accurate identification of patients likely to develop ISR. Finally, the combined application of Lp(a) and MHR yielded an AUC of 0.860 (95% CI*:* 0.731–0.989; *P* < 0.001), with specificity and sensitivity of 81.81% and 87.16%, respectively. This elevated AUC indicates that the combined assessment of Lp(a) and MHR provides a more accurate prediction of ISR occurrence, achieving high sensitivity while maintaining superior specificity, thereby outperforming each marker alone. Overall, while the individual predictive abilities of these biomarkers were limited, their combined analysis offered a more comprehensive and precise method for ISR prediction, providing valuable information for clinical screening and early intervention.

Based on the significant risk factors identified through logistic regression and GEE analyses—monocyte count, Lp(a), and MHR—a weighted risk scoring system was developed (Table 6). Regression coefficients (OR*s* or *β* values) were used as weights to generate individual patient scores, resulting in a total risk score. Specifically, the monocyte count had an OR of 1.952, indicating that each unit increase significantly raised the risk of ISR. Patients with values above the median were assigned 1 point, while those below the median received 0 points. Lp(a) had a *β* value of 1.472, reflecting an increase in ISR risk with higher levels. Accordingly, the high-level group was assigned 1 point, and the low-level group 0 points. MHR showed a stronger association with ISR, with a *β* value of 2.482; therefore, patients in the high-level group received 2 points, while those below the median were assigned 0 points. The total score ranged from 0 to 4, with higher scores indicating a greater risk of ISR.

**Table 6:** Risk scoring system for ISR based on MHR and Lp(a) in patients with DES implantation.

Risk factors	High-level group	Score	Low-level group	Score
Monocyte count	Above median	1	Below median	0
Lp(a) level	Above median	1	Below median	0
MHR	Above median	2	Below median	0
Total score	Max: 4		Min: 0	

Lp(a), lipoprotein(a); MHR, monocyte-to-HDL ratio.

**Figure 2:**
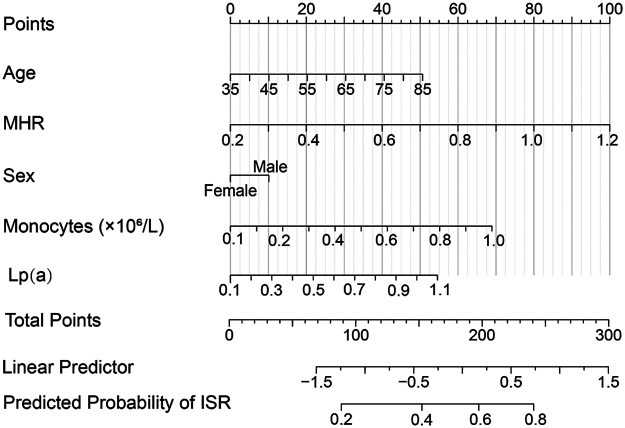
Nomogram for predicting the risk of in-stent restenosis (ISR) in coronary heart disease patients after drug-eluting stent (DES) implantation. The nomogram integrates multiple risk factors, including age, monocyte count, monocyte-to-HDL ratio (MHR), sex, and lipoprotein(a) [Lp(a)] levels. Each variable is assigned a score, and the total points are used to calculate the predicted probability of ISR. The horizontal scales represent the contribution of each factor to the total points, while the linear predictor and predicted probability of ISR are also displayed, providing a visual tool for individualized risk assessment.

At the one-year follow-up, the incidence of MACE was 12.0% (18/150) in the non-ISR group, while it was significantly higher at 30.0% (12/40) in the ISR group. The specific events included all-cause mortality, recurrent STEMI, non-fatal stroke, malignant arrhythmia, and target vessel revascularization. Although the ISR group demonstrated higher rates of all-cause mortality and recurrent STEMI, these differences did not reach statistical significance (*P* *=* 0.082 and *P* *=* 0.090, respectively). Based on the ISR risk scoring system, patients were stratified into a low-risk group (0 points) and a high-risk group (1–4 points). The results showed that the incidence of MACE was significantly higher in the high-risk group compared to the low-risk group (35% vs. 10%), suggesting that the scoring system, which incorporates MHR and Lp(a), effectively predicts the risk of MACE and serves as a valuable tool for clinical risk stratification and management. Figure 2 presents a nomogram for predicting the risk of ISR after DES implantation. This nomogram incorporates multiple risk factors, including age, MHR, sex, monocyte count, and Lp(a) levels, with each variable assigned a score. The total score is then converted into the predicted probability of ISR, providing clinicians with an individualized risk assessment tool. The contribution of each variable is visually displayed through horizontal scales and scores, allowing for a clear evaluation of a patient's specific risk based on the combination of these factors.

## 4 Discussion

This study found that patients who developed ISR following DES implantation had significantly higher levels of white blood cell count, monocyte count, MHR, and Lp(a), along with lower HDL-C levels, compared to those without ISR. Additionally, these patients had more complex coronary lesions, as indicated by higher SYNTAX scores and a greater prevalence of multivessel disease. These findings suggest that chronic inflammation, dysregulated lipid metabolism, and lesion complexity play key roles in the development of ISR. This study provides valuable evidence for the early identification of high-risk patients and the development of individualized intervention strategies. While coronary angiography (CAG) remains the gold standard for diagnosing ISR, its invasive nature limits patient compliance and broader applicability, highlighting the need for non-invasive, simple, and cost-effective risk prediction tools. The results emphasize the potential value of MHR and Lp(a) in predicting ISR, offering new insights into clinical risk stratification and personalized management.

### 4.1 Analysis of ISR risk characteristics

In this study, we found that patients with ISR had significantly higher monocyte counts, MHR, and Lp(a) levels, along with lower HDL-C levels. These findings suggest that chronic inflammation and lipid metabolism disturbances may play important roles in the development and progression of ISR. These results are consistent with previous studies (19–21). As an integrated marker of inflammation and lipid imbalance, MHR has been shown to be closely associated with coronary atherosclerosis (22, 23). Furthermore, Lp(a) contributes not only to the formation of atherosclerotic plaques but also plays a significant role in immune and inflammatory processes. Our study also observed that patients with ISR had a higher frequency of left main and multivessel disease, indicating that coronary lesion complexity may increase the risk of ISR by exacerbating vascular injury and promoting neointimal hyperplasia. Although no significant differences were found in age, BMI, or hypertension between the two groups in this study, these factors have been identified as important risk factors for ISR in previous research (24–26), and the lack of significant findings here may be due to sample size limitations or patient heterogeneity.

### 4.2 The role of MHR, Lp(a), and inflammatory Status in ISR

Inflammation plays a central role in the initiation and progression of atherosclerosis, with endothelial injury, oxidative stress, and thrombosis being key pathological mechanisms. Large-scale clinical trials, such as CANTOS, COLCOT, and LoDoCo2, have demonstrated the benefits of anti-inflammatory therapy (e.g., colchicine) in CHD, leading to its approval by the FDA in 2023 as the first anti-inflammatory treatment for CHD (27–29). In addition, statins reduce cardiovascular event risk partly due to their anti-inflammatory properties (30). Previous studies have shown that inflammatory markers such as high-sensitivity C-reactive protein (hs-CRP), neutrophil-to-lymphocyte ratio (NLR), and eosinophil cationic protein are closely associated with ISR (31), highlighting the link between inflammation and ISR as a focal point of academic interest. This study further demonstrated that patients with ISR exhibited more pronounced inflammatory features, with elevated white blood cell counts, monocyte counts, and MHR levels, which is consistent with the findings of Li et al. and others, suggesting that monocytes may serve as early predictors of ISR (32). MHR, a sensitive marker of chronic inflammation and oxidative stress, has been significantly linked to both CHD and ISR (33–35). Peng et al. were the first to identify MHR as an independent predictor of CHD (36). MHR reflects the activation status of monocytes, which are key players in vascular inflammation. Studies have shown that monocytes not only play an important role in atherosclerosis formation but also in the migration and proliferation of endothelial cells during vascular repair after injury (37). In the context of ISR, monocytes activate the vascular endothelium by secreting pro-inflammatory cytokines (e.g., TNF-α and IL-6), promoting endothelial dysfunction and smooth muscle cell proliferation, ultimately leading to vascular remodeling and restenosis (38). Therefore, as a ratio of monocytes to HDL, MHR can reflect inflammatory activity within the vasculature and may contribute to ISR development by affecting immune responses and vascular repair processes. Our results reinforce the potential role of MHR in ISR development, indicating that patients with ISR may have heightened levels of chronic inflammation and oxidative stress, thereby providing a biological basis for ISR formation. Ardahanli et al. (39) also emphasize the central role of oxidative stress in cardiovascular diseases, particularly in processes like atherosclerosis, hypertension, and myocardial ischemia-reperfusion injury, where oxidative stress increases reactive oxygen species (ROS) levels, leading to endothelial dysfunction, vascular inflammation, and cardiac remodeling. These findings align with our observations, suggesting that MHR, as a marker of oxidative stress and inflammation, plays a significant role in ISR. Regarding lipid metabolism, we found significantly lower HDL-C and higher Lp(a) levels in ISR patients, indicating a greater risk of dyslipidemia. Both low HDL-C and elevated Lp(a) are well-documented to be closely related to the progression of atherosclerosis and ISR, further supporting Lp(a) as a target for interventions aimed at endothelial injury and atherosclerosis (40–42). Lp(a), a specific subtype of low-density lipoprotein, contributes to endothelial injury and accelerates atherosclerosis by binding to inflammatory proteins, such as oxidized LDL (43) In the context of ISR, Lp(a) may exacerbate vascular inflammation by inducing endothelial damage and increasing the risk of thrombosis (44). Although the exact mechanisms of Lp(a) remain incompletely understood, its potential role in vascular repair should not be overlooked. Studies suggest that Lp(a) can influence vascular smooth muscle function by binding to receptors on endothelial cells, thereby affecting the vascular remodeling process (45).

In summary, both MHR and Lp(a) are closely related to vascular inflammatory responses and may also play an important role in the repair process after vascular injury. In the context of ISR, these two factors may interact to affect immune system responses, endothelial function, and vascular repair mechanisms, thus promoting vascular remodeling and restenosis. Therefore, future studies should further explore the specific mechanisms by which MHR and Lp(a) contribute to ISR development and assess their potential as biomarkers in risk prediction and personalized treatment. Additionally, we did not observe significant differences between groups in neutrophil, lymphocyte, or platelet counts, nor in common clinical parameters such as heart rate, blood pressure, or EF, indicating that these factors may have limited direct roles in ISR occurrence.

### 4.3 Analysis of the relationship between stent characteristics and ISR

Stent parameters, including the number of stents, stent length, deployment pressure, and diameter, have long been considered potential contributors to the risk of ISR. However, this study did not observe statistically significant differences in these characteristics between the ISR and non-ISR groups. The impact of stent number remains controversial; some studies suggest that a higher number of stents may increase vascular injury, promoting ISR (46), while others have found no significant association (17). Similarly, while longer stents are believed to increase the risk of restenosis, especially in cases of inadequate vessel expansion (47, 48), some studies report that the influence of stent length may be limited (49). Excessive deployment pressure may induce endothelial injury, potentially triggering ISR, yet this effect is influenced by multiple factors, such as patients' baseline conditions, vascular characteristics, and operator technique (50). Stent diameter is closely related to vessel anatomy, and proper matching may help reduce ISR risk; however, due to the complexity of vascular anatomy and individual variability, the exact relationship remains unclear. Although stent parameters have been widely discussed in the literature, our study did not find a significant association between these parameters and ISR occurrence. Several factors may contribute to this result. First, while stent parameters may influence ISR, their effects could be modulated by other factors, including the patient's underlying diseases, inflammatory status, and medication regimen. In our study, the types and intensities of medications (such as statins and PCSK9 inhibitors) were not strictly controlled, which may have obscured the relationship between stent characteristics and ISR. Second, technical factors, such as stent placement accuracy and individual vascular anatomy, may vary across patients. These factors were not fully accounted for in this study, which could have influenced the results. Therefore, the impact of stent parameters on ISR may have been confounded by other variables, leading to no statistically significant association. Overall, our findings suggest that individual stent parameters alone may be insufficient as independent predictors of ISR. The occurrence of ISR is likely the result of multiple interacting factors, including patients' inflammatory status, lipid metabolism abnormalities, and genetic predisposition. Future multi-center, large-scale studies are needed to validate these findings and explore the development of comprehensive predictive models that integrate stent characteristics with biomarkers and clinical risk factors to enhance ISR risk assessment and facilitate individualized treatment strategies.

### 4.4 Predictive value of monocytes, Lp(a), and MHR in ISR

This study found that monocyte count, Lp(a), and MHR were closely associated with the occurrence of ISR, reinforcing the critical roles of inflammation and lipid metabolic abnormalities in the development of ISR. Monocytes contribute to ISR risk by mediating inflammatory responses, infiltrating the vessel wall, and promoting the formation and instability of atherosclerotic plaques, a mechanism supported by multiple studies (5). Lp(a), a unique lipoprotein, is thought to increase ISR incidence by promoting endothelial injury, enhancing pro-inflammatory responses, and facilitating thrombogenesis (17, 42). Elevated Lp(a) levels have been consistently linked to CHD, stroke, and other cardiovascular events. Furthermore, MHR, a composite marker reflecting both inflammatory activity and lipid metabolic disturbances, has demonstrated significant predictive value for CHD and vascular events (23, 33, 34). In this study, both GEE and multivariate logistic regression analyses confirmed monocyte count, Lp(a), and MHR as independent risk factors for ISR. Notably, ROC curve analysis demonstrated that all three biomarkers—monocyte count, Lp(a), and MHR—exhibited strong predictive capabilities for ISR. Among these, Lp(a) was particularly effective at identifying low-risk patients, whereas MHR demonstrated higher sensitivity for screening high-risk individuals. Combining Lp(a) and MHR for detection significantly improved both sensitivity and specificity, suggesting that their joint assessment could provide a more accurate tool for ISR risk evaluation in clinical practice. Overall, the results of this study further emphasize the potential clinical value of monocyte count, Lp(a), and MHR in predicting ISR risk, aiding in the early identification of high-risk patients and facilitating personalized interventions. However, further multi-center, large-scale, prospective studies are required to confirm the predictive efficacy of these biomarkers and explore their broader application in cardiovascular disease risk stratification. As medical technology continues to evolve, artificial intelligence is becoming increasingly important in the early diagnosis of cardiovascular diseases. Arıkan et al. (51) discussed the potential of artificial intelligence in diagnosing cardiac dysfunction in the emergency department, highlighting that AI technology can significantly improve the diagnostic accuracy of cardiac ultrasound (POCUS), especially in clinical settings where quick and accurate decisions are required. This perspective further supports the analysis of inflammation markers and cardiovascular disease prediction in this study, suggesting that the integration of AI technology could further optimize early diagnosis and risk assessment for ISR.

### 4.5 Analysis of the relationship between MACE and the risk scoring system

MACE is a key clinical endpoint in cardiovascular disease, encompassing all-cause mortality, myocardial infarction, stroke, and other adverse events. During follow-up, we observed that the incidence of MACE in the ISR group was significantly higher than in the non-ISR group (30% vs. 12%), indicating that the occurrence of ISR is closely related to cardiovascular event risk. Similar studies have also shown that the occurrence of ISR often serves as a marker for poor cardiovascular outcomes, particularly in patients undergoing DES implantation (52). Our risk scoring system, which combines the biomarkers MHR and Lp(a), both related to inflammation and lipid metabolism, effectively predicts the risk of ISR and further helps identify high-risk MACE patients. This finding is consistent with previous research, indicating that a combined assessment of inflammatory markers (such as MHR) and lipid metabolic markers [such as Lp(a)] is more accurate than using a single indicator in predicting long-term MACE and mortality (53). Future studies should further validate the applicability of these biomarkers in different populations and explore their prognostic value in multicenter, large-scale studies. Additionally, integrating other clinical features and advanced imaging techniques could further optimize the risk assessment models for ISR and MACE, improving individualized clinical management.

### 4.6 Limitations

Although this study provides valuable data on the relationships of monocyte count, Lp(a), and MHR with ISR, several limitations should be acknowledged. First, the sample size imbalance between the ISR group and the Non-ISR group may affect statistical power and increase the risk of type II errors. This imbalance could lead to biased interpretation of the results, thereby limiting the external generalizability of the conclusions. Ardahanli et al. (54) recently pointed out that the limitations inherent to observational studies and small sample sizes may compromise the interpretation of causal relationships, and that potential confounding factors such as heart failure were not adequately taken into account. These issues suggest that, when designing similar studies, it is essential to consider such potential confounders and to strengthen the comprehensiveness and accuracy of the data. Second, although various biomarkers associated with ISR were investigated, this study did not systematically incorporate other important factors that could influence ISR risk, such as patients' genetic backgrounds and lifestyle habits. Third, while ROC curve analysis demonstrated that combining MHR and Lp(a) significantly enhanced predictive performance, the sensitivity and specificity of these markers may still be affected by sample composition and measurement methodologies, necessitating further validation in larger, multicenter cohorts. Another limitation of this study is that the inclusion and exclusion criteria did not account for the type, intensity, or duration of lipid-lowering therapy (LLT), particularly statins and PCSK9 inhibitors. These medications not only lower lipid levels but also possess anti-inflammatory properties (55, 56), which may affect inflammatory markers such as monocyte count and MHR, thereby confounding the relationship with ISR. Future studies should consider incorporating the type, intensity, and duration of lipid-lowering therapy in the analysis to more accurately assess its impact on ISR risk. Finally, although this study focused on inflammatory and lipid metabolic markers, it did not delve into the interactions of these biomarkers with vascular repair processes, cytokines, and other potential pathological mechanisms. Future research should integrate molecular biology and multi-omics approaches to elucidate their specific mechanistic roles in ISR development. In conclusion, while this study highlights the potential application value of monocyte count, Lp(a), and MHR in predicting ISR risk, large-scale, prospective studies are still required to further validate these findings and ensure their reliability and broader applicability in clinical practice.

## 5 Conclusion

This study demonstrated that monocyte count, Lp(a), and MHR are strongly associated with the occurrence of ISR in CHD patients following DES implantation. The combination of MHR and Lp(a) showed robust predictive value for ISR. These findings provide new clinical insights to aid in the early identification of high-risk patients after PCI and support the development of individualized management strategies, including intensified anti-inflammatory therapy. Such approaches may help reduce the incidence of restenosis and improve long-term patient prognosis. Future large-scale, prospective studies are needed to validate these results and further refine their application in clinical risk stratification and precision medicine.

## Data Availability

The raw data supporting the conclusions of this article will be made available by the authors, without undue reservation.
